# Dr. John Duncan MacPherson

**Published:** 1912-12

**Authors:** 


Dr. John Duncan MacPherson of Akron, N. Y., Michigan, 1883, died at his home, Oct. 31, 1912.

